# Facebook as a source of access to medicines

**DOI:** 10.1371/journal.pone.0275272

**Published:** 2022-10-13

**Authors:** Tomasz Zaprutko, Dorota Kopciuch, Anna Paczkowska, Józefina Sprawka, Julia Cynar, Monika Pogodzińska, Katarzyna Niewczas, Aleksandra Stolecka, Maria Sygit, Michał Michalak, Piotr Ratajczak, Krzysztof Kus

**Affiliations:** 1 Department of Pharmacoeconomics and Social Pharmacy, Poznan University of Medical Sciences, Poznan, Poland; 2 Student Scientific Society, Department of Pharmacoeconomics and Social Pharmacy, Poznan University of Medical Sciences, Poznan, Poland; 3 Department of Computer Sciences and Statistics, Poznan University of Medical Sciences, Poznan, Poland; Linnaeus University: Linneuniversitet, SWEDEN

## Abstract

Over the last decade, we’ve observed an enormous social media development, which have recently become commercial services. Hence, we aimed to verify if Facebook might be considered as a source of access to medicines. We also intended to identify prescription (Rx) and over the counter (OTC) medicines if available on that platform. The study was conducted from January 2019 to July 2020. We investigated offers of pharmaceuticals posted on Facebook (individual posts and communities) between 2016 and 2019. Medicines were divided into Rx and OTC brands and in accordance with their possible therapeutic use. We verified whether the medicines were for sale, to exchange, or available for free. Posts and groups were investigated for example, by entering the phrase “for free,” followed by a city in Poland. Out of 409 offers, 380 (92.91%) concerned Rx brands and 29 (7.09%) OTC brands. 315 (77.02%) medications were available “for free”. In 47 (11.49%) cases, an “exchange” was expected, and 47 (11.49%) announcements were marked as “for sale”. Cardiovascular medicines (n = 125; 30.56%) were the most popular. There were also antidiabetics (n = 38; 9.29%) and antipsychotics (n = 28; 6.84%). We also found clear candidates for misuse. These were opioids (n = 7; 1.71%), benzodiazepines (n = 2; 0.48%), clomifene (n = 1; 0.24%), and letrozole (n = 1; 0.24%). Facebook provides the possibility of uncontrolled access to medicines. The majority of offers concerned Rx brands, including opioids and benzodiazepines. Medicines offered on Facebook were mainly accessible for free. The scope and the number of medications offered on Facebook should make this issue a hazardous phenomenon.

## Introduction

The Internet was designed to be unstoppable, and essentially it is [1]. It contributes, among others, to the enormous development and popularity of online communities, networks, and social media like Facebook or Twitter. These services have become the primary source of general information, especially for teenagers and young adults [2, 3], including information on health and medicines [2, 3].

Nevertheless, medicines are not the same commodities like clothes or office supplies, for instance [4]. Despite many indisputable benefits related to pharmacotherapy, medications might be harmful when not used correctly [5]. Hence, most of them require a prescription or advice from trained medical professionals or pharmacists before they can be taken [4]. In online reality, drugs are frequently marketed and sold as “no prescription required” and without risk-related information of possible side effects or interactions [6]. This contributes to the significant scope of falsified medicines available on the Internet [6–8]. Considering that drug misuse has become a global public health and economic issue [7, 9, 10], local and international agencies should focus on decreasing the risks of buying medicines over the Internet platforms [11] or social networking services which like Facebook do not have a medicines license. Despite the swift growth of the trade of several goods on Facebook it is, to our knowledge, the first study evaluating Facebook as a platform allowing access to medicines.

For instance, in the European Union (EU), patients should only buy medicines from online pharmacies registered with the EU Member States’ competent national authorities. Besides, the European Commission has imposed a common logo on these registered retailers [12]. In the USA, one of the United States Food and Drug Administration’s (FDA) recommendations for safe online pharmacies is to have a U.S. state-licensed pharmacist available to answer your questions. Moreover, the patient should check on the pharmacy’s website for the seal of the National Association of Boards of Pharmacy (NABP) [13].

Facebook facilitates sharing information, messages, and the exchange of goods [14]. The variety of features that serve different purposes for the user also provide health education [14, 15]. Therefore social media platforms have increasingly enabled drug manufacturers to engage with patients and healthcare providers [15]. Simultaneously, however, social media are being redesigned to improve their commercial services, which allow for selling or exchanging commodities, including medicines. The popularity, lack of control, and global access to information and goods shared by social media make medicines’ trade on such services a hazardous issue.

Our study aimed to verify if Facebook might be considered as a source of access to medicines. We also intended to evaluate the scope of that phenomenon and to identify prescription (Rx) and over the counter (OTC) medicines, if available.

## Materials and methods

The study was conducted from January 2019 to July 2021 and both data collection and analysis complied with conditions for the source of the data. The study period also covers the start of the COVID-19 pandemic, which contributed to many people’s financial insecurity and related difficulties. Being in crisis, some started their activity with digital currency exchange platforms, betting, playing in the stock exchange, or online trade. Hence, it is conceivable that medicines could have been used for that purpose, and people used social media sites like Facebook for such trade.

**Fig 1:**
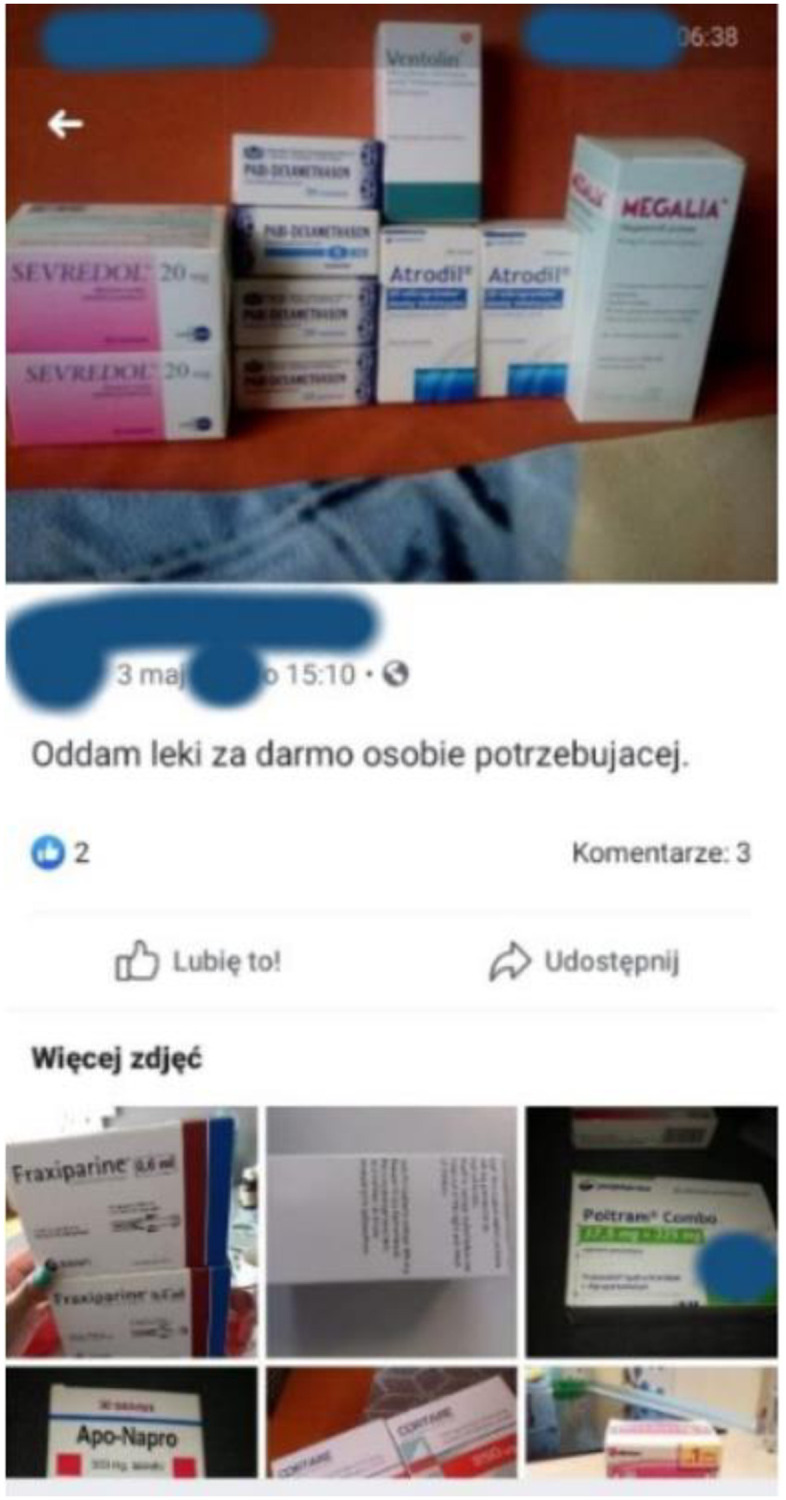
The example of the illegal online trade of medicines. Offer including morphine (Sevredol^®^) and tramadol (Polram Combo^®^). Source: Facebook. The URL address was not collected and is not reported to avoid the possible traceability of the Facebook user and to not to contravene the data protection or the GDPR. The presented figure is similar but not identical (partial screenshot with concealed parts of the picture) to the original image and is therefore only for illustrative purposes.

**Fig 2:**
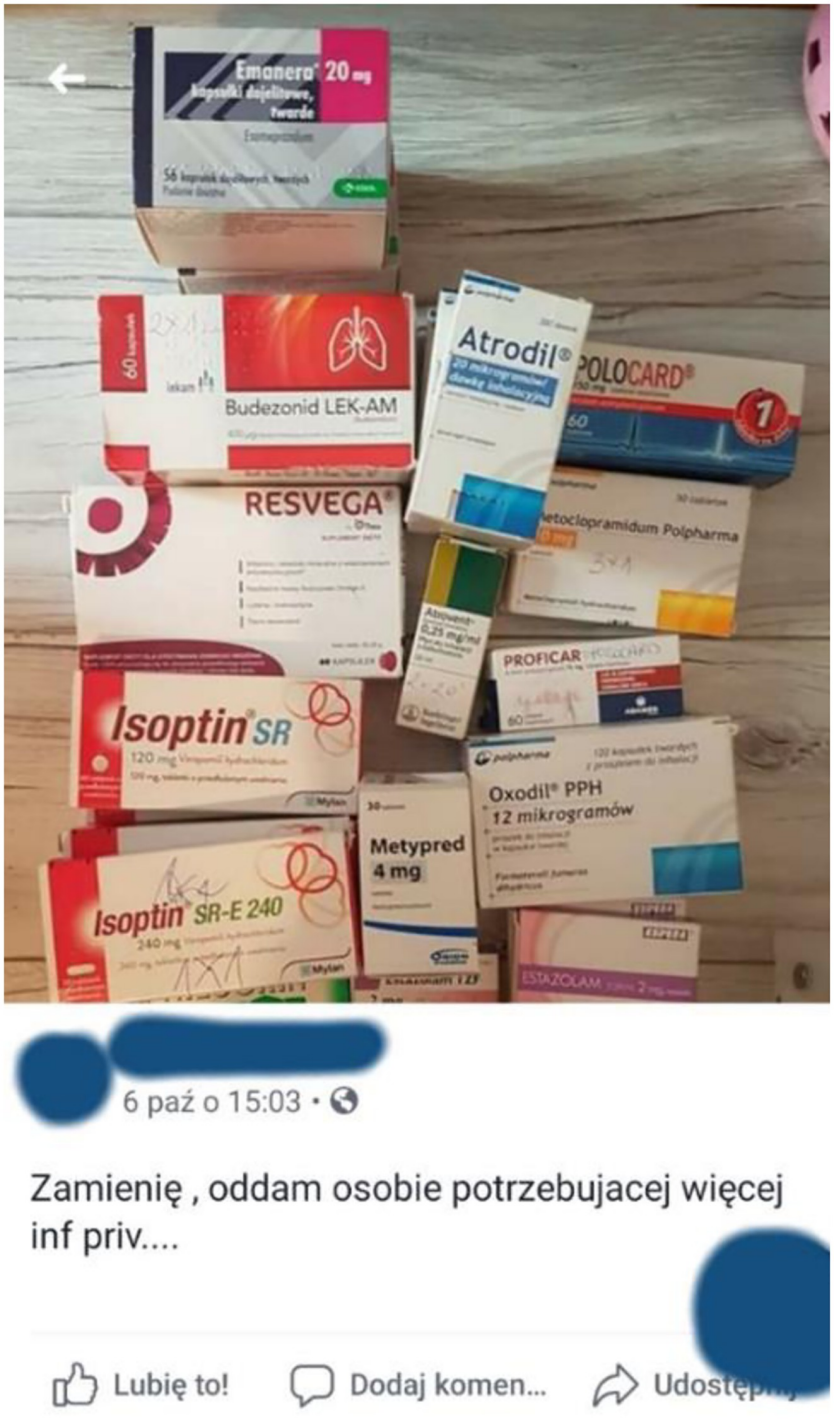
The example of the illegal online trade of medicines. Offer for estazolam (Estazolam^®^). Source: Facebook. The URL address was not collected and is not reported to avoid the possible traceability of the Facebook user and to not to contravene the data protection or the GDPR. The presented figure is similar but not identical (partial screenshot with concealed parts of the picture) to the original image and is therefore only for illustrative purposes.

**Fig 3:**
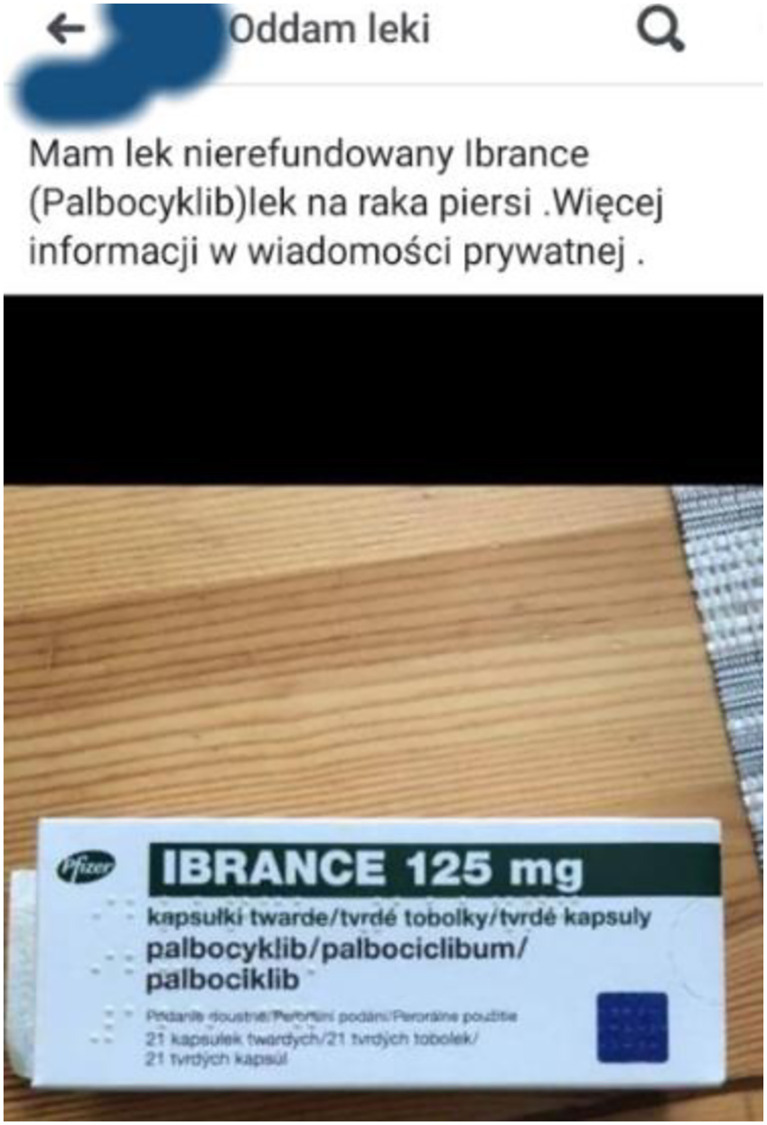
The example of the illegal online trade of medicines. Offer for palbociclib (Ibrance^®^). Source: Facebook. The URL address was not collected and is not reported to avoid the possible traceability of the Facebook user and to not to contravene the data protection or the GDPR. The presented figure is similar but not identical (partial screenshot with concealed parts of the picture) to the original image and is therefore only for illustrative purposes.

We investigated, written in Polish, offers of pharmaceuticals posted on Facebook (individual posts and communities) from 2016 to 2019. Out of 409 trade offers found in that period, 13 were from 2016, 23 concerned 2017, 111 concerned 2018, and 262 concerned 2019. We examined these offers to verify if medicines were for sale, to exchange for other goods, or available for free. We investigated posts and groups using the Facebook browser, for example, by entering the phrase “for free” followed by a city in Poland, such as Poznan or Warsaw. We used our private accounts to mine the data, joining open and, if accepted by the moderator, closed groups or societies on Facebook like e.g., “zero waste” followed by the city name. We have investigated posts only from private accounts excluding shops, retail points, or anonymous offers. If an offer of medicines trade was found, we saved just information in the excel sheet about the medicine and expected payment form. No personal data was collected so as not to contravene the data protection or the GDPR [16]. We have not contacted tenderers to be not accused of having personal data in the contact box. In case of screenshots (Figs 1–3) confirming, in our opinion, the most important examples of medicines traded on Facebook, we anonymized all posts permanently removing or hiding personal information. For that purpose, we used Corel Photo-Paint 9. Besides, we collected data only for scientific purposes. In the case of screenshots and figures, these were similar but not identical (partial screenshot with concealed parts of the picture) to the original image and, therefore, only for illustrative purposes. Moreover, we obtained the confirmation from the Bioethics Committee that the study is not a medical experiment and, according to the Polish law and GCP regulations, does not require approval of the Bioethics Committee at Poznan University of Medical Sciences.

Medicines were divided into Rx and OTC brands and in accordance with their possible therapeutic use. Because of the scope of investigated medicines and the fact that the majority of the offers concerned Rx brands, we decided to present medications categorized in the following groups: antibiotics, anticancer medicines, anticoagulants, antidiabetics, anti-asthma medications, antihistamines, antipsychotic medicines, cardiovascular medicines, hormones, and painkillers. We classified the rest of the medicines into the group “others”.

In our opinion there were substances which might be categorized in several groups like novel oral anticoagulants (NOACs) in cardiovascular medicines or anticoagulants. Nonetheless, these medicines like rivaroxaban, dabigatran, apixaban were classified into the cardiovascular medicines category. Anticoagulants, however, were dedicated to substances like enoxaparin, for instance. We decided to classify neuroleptics, antidepressants, and benzodiazepines as antipsychotic medicines. Medicines found severally or very rarely like immunosuppressant’s or veterinary medicines were classified as “others”.

The use of brand names is strongly limited to avoid any accusations of advertising, although the names are sometimes presented to ensure text clarity.

Money values were converted from Polish Zloty (PLN) to EURO (€) at the average € exchange rate in July 2020 as published by the National Bank of Poland (€ 1 = PLN 4.4528). The study’s monetary values comprise rounded calculated amounts resulting from converting monetary units into the common European currency. Results presented in € should make this paper clear and useful for the readers.

## Results

Out of 409 offers of medicines posted on Facebook, 380 (92.91%) concerned Rx brands, and 29 (7.09%) OTC. 315 (77.02%) medications were available “for free”. Both offers marked as “for exchange” and “for sale” concerned 47 (11.49%) cases. In the group “for sale” there were 14 offers presenting the exact price ($\bar{x}$ = € 16.44) and the rest was negotiable.

We present the results with percentages in square brackets. In most cases, these are part of all analyzed offers. If the result constitutes the percentage of cases in the separated group, it is always indicated in the text. We believe that the results presented in that way will contribute to the clearness of this chapter.

### Types of medicines found

The most popular medicines were cardiovascular medicines (n = 125; 30.56%), including eight offers (1.95%) related to novel oral anticoagulants. These were followed by antidiabetics (n = 38; 9.29%, e.g., insulins or metformin), anti-asthma medications (n = 30; 7.33%, e.g., ciclesonide, budesonide), and antipsychotics (n = 28; 6.84%, e.g., olanzapine, quetiapine). Out of 26 (6.35%) offers for hormones the majority (n = 15; 3.66%) were related to contraceptives. The one offer (0.24%) considered clomifene and one (0.24%) was related to letrozole.

For antibiotics (e.g., amoxicillin, lymecycline) and painkillers (including opioids), there were 19 (4.64%) offers in each group. There were 17 (4.15%) posts related to anticoagulants (e.g. enoxaparin, dalteparin) and 13 (3.17%) to antihistamines (e.g., cetirizine, loratadine). 87 (21.27%) posts were in the “others” group, including three (0.73%) offers of immunosuppressant’s (e.g., ciclosporin), and two (0.48%) of veterinary medicines (e.g., doxycycline for dogs). One (0.24%) offer from the “others” group considered Mysimba^®^ (€ 77.04), which is the Rx brand used among obese and overweight patients. It contains, however, naltrexone and bupropion, which are individually used in the treatment of alcoholism and depression, respectively [17]. With reference to the medicines groups, results are also presented in Table 1, which was created to provide transparency for those who prefer tabulated data.

**Table 1:** The examples of medicines found on Facebook^#^.

Medicines from Facebook	Medicines category	Number of advertised medicines	Number of medicines offered „for sale”	Example of the found medicine (brand name/substance/ATC Code)	Price of presented example in Euro (€)
409 offers found in the study^$^	Cardiovascular including novel oral anticoagulants	n = 125 (30.56%)	3	Dopegyt^®^ / 250 mg Methyldopa / C02AB01	€ 6.74
	Hormones	n = 26 (6.35%)	4	Duphaston^®^ / 10 mg Dydrogesterone / G03DB01	€ 3.37
	Anticoagulants	n = 17 (4.15%)	2	Clexane^®^ / 40 mg enoxaparin sodium / B01AB05	€ 3.37
	Antihistamines	n = 13 (3.17%)	1	CetAlergin^®^ / 10 mg cetirizine dihydrochloride / R06AE07	€ 1.12
	Others	n = 87 (21.27%)	4	Mysimba^®^ / 8mg naltrexone hydrochloride+ 90 mg bupropion hydrochloride / A08AA62	€ 78.60

^#^We present only these groups of medicines where the exact retail price was indicated.

^$^The number concerns all analyzed offers for medicines.

### Types of exchange agreement

Despite that the majority of investigated medicines were “for free,” there were also trade offers. For instance, out of 19 offers in the group of antibiotics there were 15 (78.95%) posts “for free”, 1 (5.26%) for exchange, and 3 (15.79%) offers for sale in that group. Considering painkillers (including opioids), out of 19 in that group there were 17 (89.47%) posts for free and 2 (10.53%) for sale. The structure of offers related to opioids, benzodiazepines, and anti-cancer medicines is presented in Table 2. We showed these relatively small groups of studied medicines separately, because of their therapeutic significance and risk of a recreational use.

**Table 2:** The structure of offers related to opioids, benzodiazepines, and anti-cancer medicines^#^.

	Opioids n = 7			Benzodiazepines n = 2		Anti-cancer medicines n = 7				
	Morphine	Tramadol	Oxycodone	Estazolam	Zolpidem	Megestrol	Palbociclib	Ondansetron	Filgrastim	Letrozole
	n = 2	n = 4	n = 1	n = 1	n = 1	n = 2	n = 2	n = 1	n = 1	n = 1
**For free**	n = 2	n = 4	n = 1	n = 1	n = 1	n = 2	n = 2	n = 1	n = 1	N/A
**For sale**	N/A	N/A	N/A	N/A	N/A	N/A	N/A	N/A	N/A	n = 1^^^

N/A–not applicable.

^#^ There were no offers for the exchange within these groups of medicines.

^ Negotiable price.

**Fig 4:**
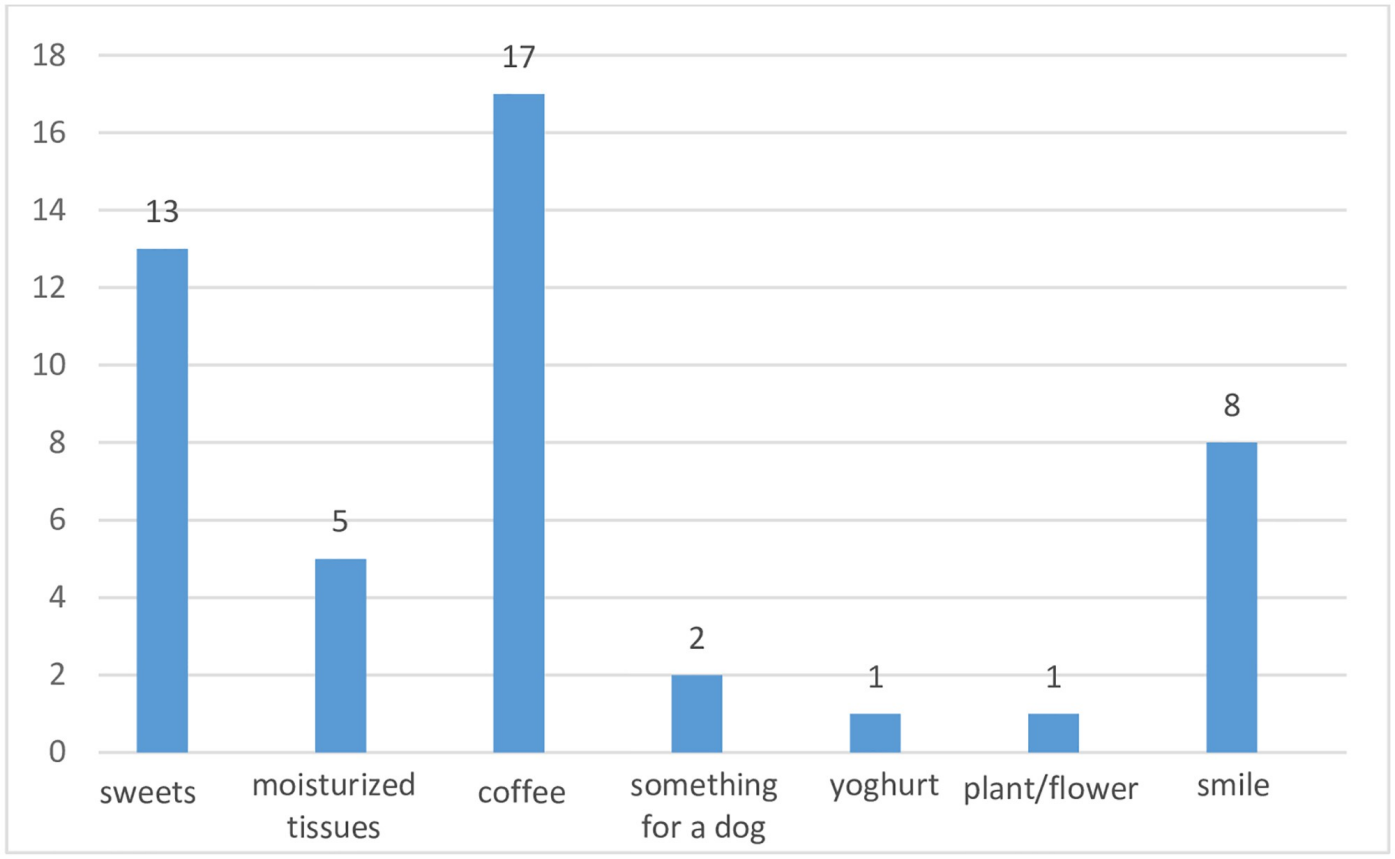
Goods expected in exchange for medicines.

Among goods expected for exchange, the most popular was coffee, followed by sweets and a smile. Although a smile might be categorized in the group “for free”, we decided to analyze it among the "exchange" group, emphasizing the importance of the studied phenomenon. Besides, the smile was clearly indicated as an expected commodity. The type of goods may result from the characteristics of market places like Facebook or similar platforms, where people frequently offer goods within local societies in exchange for something symbolic. The structure of goods expected by the bidders is presented in Fig 4. In the group of posts offered for exchange, we found, e.g., olanzapine, dabigatran, and rovamycine.

In the case of medicines “for sale”, the highest price (€ 96.57) pertained to SYNVISC ONE^®^HYLAN G-F 20, which is osteoarthritis knee pain relief [18]. The cheapest was the OTC cetirizine (€ 1.10). If a medicine was “for sale” the usual offer ranged between € 3.37 and € 6.74. However, in 29 cases, the payment method was to be negotiated. It concerned, e.g., clomifene and letrozole, which are clear candidates for misuse. We also found one offer of lutein for € 3.37, which pregnant women frequently use. Hence the usage should be controlled by professional medical staff instead of self-treatment.

Interestingly, there was one offer for 5 mg bisoprolol 28 pills (Bisocard ^®^) for € 3.37 likewise. In community pharmacies, this medicine is usually cheaper and costs between € 2.25 and € 3.00. Nevertheless, the brand mentioned above is not reimbursed in Poland. Thus the price may vary between pharmacies and as presented in the study, the medicine might be cheaper buying it in the regular pharmacy than on Facebook.

Among the offered medicines, there were also medicines requiring special storage conditions like filgrastim or insulins. Nevertheless, posts and the content of the offers did not confirm suitable storage and further transportation. We also found one case of medicine that is not officially available in Poland (SabSimplex^®^ against infantile colic).

## Discussion

### Medicines trade on Facebook might be a developing phenomenon

The analysis revealed that Facebook might be a source of uncontrolled access to several medications. Out of 409 investigated offers, the majority (92.91%) related to Rx brands. Cardiovascular medicines were most frequently (30.56%) posted on Facebook and were followed by antidiabetics (9.29%), and anti-asthma medications (7.33%). Importantly, we also found offers of medicines that might be misused. There were, among others, 7 (1.71%) posts related to opioids. Most of the investigated medicines were available for free.

The severity of medicines offered on Facebook makes this most popular networking platform an important medicines trade source. Free and uncontrolled access to medicines might be related to possible misuse of prescription drugs, which became a hazardous phenomenon worldwide [3, 9, 19, 20]. It is in line with our study, where most investigated posts considered Rx brands. Thus the development of that market may significantly jeopardize public health and boosts related costs [20, 21]. In 2017, approximately 18 million people misused prescription medicines. It mainly concerned adolescents and young adults [9]. Worldwide, opioids remain the most frequently misused prescription drugs. In the United States, the number of people who misused these medicines in 2017 was 2 million. However, in Europe, about 40% of all medicine use comprised tranquilizer and sedative misuse [9]. It contributes to the huge economic burden of that issue [20–22], and e.g., total US societal costs of prescription opioid misuse were estimated at almost $ 56 billion in 2007, influencing productivity loss, costs of health care and criminal justice [20]. Moreover, direct health care costs per person were almost nine times higher among opioid abusers ($ 15,854) than in the non-abusers group ($ 1,830) [23]. In total, however, the misuse or the use of doubtful or substandard medicines are responsible for a substantial economic burden, which in 2002 amounted to more than $ 180 billion in the USA, and in 2018 these costs rose to over $ 600 billion [23, 24].

### Unregulated access to medicines on online platforms may contribute to the misuse of medications or mislabelled medicines

It is estimated that in the USA, 36 million Americans have bought medicines online, including lifestyle and lifesaving drugs, without a valid prescription [7]. Facebook might be a source of these medicines too. In our study we revealed posts offering cardiovascular medicines, antibiotics, and antidiabetics, for example. In the case of antibiotics, there were offers for doxycycline. It could be related to the self-medication practices to manage COVID-19 [25, 26]. As a relatively cheap and easily accessible medicine, doxycycline was frequently used in the first days of the symptoms of a SARS-CoV-2 infection, especially at the beginning of the pandemic [27]. Later studies found that it should not be used as a routine treatment for COVID-19 [28]. However, it could be a more developed phenomenon than presented in this study. It might result from the underestimated number of SARS-CoV-2 infections related to limited test availability in the first months of the pandemic and then hidden cases which are frequently not reported in official statistics [29, 30].

We also found offers e.g., for morphine, oxycodone, and benzodiazepines. People build up a tolerance to these medicines very quickly, and often escalate their dosage to get a similar state. Therefore, the overdose risk is vast [4], and uncontrolled access, and misuse of these medicines may contribute to dire consequences to patients and society [4, 23, 31]. Besides, the same or very similar risks are related to the other medicines. For instance, Mysimba^®^, revealed in the study too, seems to require monitoring through pharmacovigilance stages and the safety of supply chains. Likewise, it should be an issue for all market stakeholders, including politicians and marketing authorisation holders.

We found, in our study, several offers of neuroleptics and hormones likewise. The importance of using medicines from legitimate supply chains also confirms the example from the Democratic Republic of Congo. 11 children died there and it was disclosed that they had been given tablets labeled diazepam. Still, when tested, the pills were found to contain haloperidol [32]. The trade on Facebook does not protect potential users from such dangers. In the hormones group, we mainly found contraceptives, but there were offers for clomifene and letrozole. These might be used for anabolic purposes or to counter the side effects of anabolic steroid abuse [33, 34].

Surprisingly, OTC medicines’ offers constituted a minority in our study. Still, their misuse should not be downplayed because it is also a developing problem and a serious global public health challenge [9, 10, 35]. In supra-therapeutic doses, substances like dextromethorphan or pseudoephedrine can elicit psychoactive effects such as euphoric or dissociating [9, 10]. Although pharmaceutical law in Poland was amended to reduce recreational use of OTC medicines, access to medications containing substances mentioned above is still relatively free [10]. It may explain why these and other OTC medicines were not frequently offered on Facebook.

### Internet is a source of health information and goods, including medicines

For many people, the Internet, including social media, became a primary and trustworthy source of health information [2, 36]. Nevertheless, such trust might be dangerous. It raises e.g., from the fact that online sources of information are not supported by professional information. Pharmacists as patient’s advisors guarantee drug safety and public awareness resulting, e.g., from pharmaceutical care, pharmacists’ responsibilities, and conditions aimed at suitable drug storage and delivery [37–39]. Moreover, Coloma et al. [36] revealed that data from social networks might not include complete health information, which could be essential for a correct understanding of widely commented health issues like vaccination or side effects [36, 40]. Besides, Coloma et al. [36] revealed that the country where posts originate could not be automatically identified in almost 60% of cases. It confirms the potential risks of improper storage and transportation like in the case of found insulins or filgrastim, which require cool storage conditions. It, and the study as a whole, also demonstrates some failures of the falsified medicines directive, which applies to legitimate medicines providers instead of illegal points of frequently substandard medicines turnover [39, 41]. As also stated by the Reviewer (thank you for the valuable comments), it confirms the urgent need for law amendments and the healthcare decision makers’ focus on societal education and access to safe pharmaceuticals.

The globalization of several markets and COVID-19 pandemic lead to the very fast growth of e-commerce. It also concerns the pharmaceutical market with easy purchase and delivery of medicines via the Internet [42]. Despite worldwide warnings related to the dangers of buying pharmaceuticals online, patients increasingly turn to online retailers to reduce out-of-pocket costs and to avoid queues as well as coverage-based limitations [6]. For these reasons, people look for the best retail offers, and therefore drugs are increasingly bought from informal pharmacies or websites that look legitimate, but in fact, are falsified [3, 6, 7, 32, 39, 41, 42].

The simultaneous development of medicines trade on Facebook and other Internet channels makes uncontrolled access to medicines, misuse of drugs, and their inappropriate supply an urgent issue requiring the engagement of healthcare decision-makers, politicians, and all stakeholders of the pharmaceutical market. Moreover, there is a lack of not only societal but also school education concerning risks of misuse of medicines and their access through illegitimate sources like Facebook. This social media is one of the most popular platforms among teenagers, and the uncontrolled access to several medicines from Facebook only adds to the scale of the problem.

## Limitations

The study has some limitations. Namely we did not attempt to buy the investigated medicines to evaluate bargaining possibilities or to ask about storage conditions for example. It could be interesting to check these medicines, every tenth for instance, in a laboratory to evaluate if the medicines were fake. It could be valuable to analyse not only Facebook, but also several auction sites, where medicines might be offered too. Nevertheless, this might be a topic for future research in the field. It would also be worth considering the potential advantages of the medicines market on Facebook and related networking platforms. These may provide an unrestrained chance to find a medicine that is not commonly accessible, for example, due to drug shortages.

## Conclusions

Facebook provides the possibility of uncontrolled access to medicines. The majority of offers concerned several Rx brands, including opioids and benzodiazepines. Medications offered on Facebook were mainly available for free, resulting in open access to many substances for end-users at any age. There are no guarantees of proper storage conditions, supply chain, or drug origin. The scope and the number of medicines offered on Facebook should make this issue a hazardous phenomenon. Safeguarding the pharmaceutical market and society in the age of globalization represents an urgent challenge for politicians, researchers, and all market stakeholders. Further studies are needed to aggregate data in the field and to extend the conclusions.

## References

[1] Shabot MM. Medicine on the Internet. Proc (Bayl Univ Med Cent). 2001;14: 27–31. Available: https://www.ncbi.nlm.nih.gov/pmc/articles/PMC1291308/ doi: 10.1080/08998280.2001.11927727 16369583 PMC1291308

[2] Song H, Omori K, Kim J, Tenzek KE, Hawkins JM, Lin W-Y, et al. Trusting Social Media as a Source of Health Information: Online Surveys Comparing the United States, Korea, and Hong Kong. J Med Internet Res. 2016;18. doi: 10.2196/jmir.4193 26976273 PMC4810010

[3] Nielsen S, Barratt MJ. Prescription drug misuse: is technology friend or foe? Drug Alcohol Rev. 2009;28: 81–86. doi: 10.1111/j.1465-3362.2008.00004.x 19320680

[4] Emma Hammett. Online Drug Addiction & Misuse: new rules to combat misuse. In: Online First Aid [Internet]. 9 Apr 2019 [cited 9 Jul 2020]. https://onlinefirstaid.com/online-drug-addiction/

[5] mckesson-europe-otc-medicines-sales-data-data.pdf. https://www.mckesson.eu/mck-en/resource/blob/24540/7a53568c088bf726ba088a7c722b26d7/mckesson-europe-otc-medicines-sales-data-data.pdf

[6] Liang BA, Mackey TK, Lovett KM. Illegal “no prescription” internet access to narrow therapeutic index drugs. Clin Ther. 2013;35: 694–700. doi: 10.1016/j.clinthera.2013.03.019 23597722

[7] Blackstone EA, Fuhr JP, Pociask S. The Health and Economic Effects of Counterfeit Drugs. Am Health Drug Benefits. 2014;7: 216–224. Available: https://www.ncbi.nlm.nih.gov/pmc/articles/PMC4105729/ 25126373 PMC4105729

[8] Vida RG, Fittler A, Mikulka I, Ábrahám E, Sándor V, Kilár F, et al. Availability and quality of illegitimate somatropin products obtained from the Internet. Int J Clin Pharm. 2017;39: 78–87. doi: 10.1007/s11096-016-0398-y 27888454

[9] Siste K, Nugraheni P, Christian H, Suryani E, Firdaus KK. Prescription drug misuse in adolescents and young adults: an emerging issue as a health problem. Curr Opin Psychiatry. 2019;32: 320–327. doi: 10.1097/YCO.0000000000000520 31045614

[10] Zaprutko T, Koligat D, Michalak M, Wieczorek M, Józiak M, Ratajczak M, et al. Misuse of OTC drugs in Poland. Health Policy. 2016;120: 875–881. doi: 10.1016/j.healthpol.2016.06.008 27344198

[11] Risks of buying medicines over the internet. In: nidirect [Internet]. 9 Nov 2015 [cited 9 Jul 2020]. https://www.nidirect.gov.uk/articles/risks-buying-medicines-over-internet

[12] European Medicines Agency. Buying medicines online. In: European Medicines Agency [Internet]. 17 Sep 2018 [cited 29 Jul 2020]. https://www.ema.europa.eu/en/human-regulatory/overview/public-health-threats/falsified-medicines/buying-medicines-online

[13] U.S. Food and Drug Administration. Commissioner O of the. How to Buy Medicines Safely From an Online Pharmacy. FDA. 2019 [cited 29 Jul 2020]. https://www.fda.gov/consumers/consumer-updates/how-buy-medicines-safely-online-pharmacy

[14] Ventola CL. Social Media and Health Care Professionals: Benefits, Risks, and Best Practices. P T. 2014;39: 491–520. https://www.ncbi.nlm.nih.gov/pmc/articles/PMC4103576/PMC4103576 25083128

[15] Research C for DE and. For Industry: Using Social Media. FDA. 2020 [cited 24 Jul 2020]. https://www.fda.gov/about-fda/center-drug-evaluation-and-research-cder/industry-using-social-media

[16] D20181000Lj.pdf. https://isap.sejm.gov.pl/isap.nsf/download.xsp/WDU20180001000/U/D20181000Lj.pdf

[17] Mysimba 8 mg/90 mg prolonged-release tablets—Summary of Product Characteristics (SmPC)—(emc). [cited 31 Mar 2022]. https://www.medicines.org.uk/emc/product/2684#gref

[18] SYNVISC ONE^®^ HYLAN G-F 20 Prescribing Information. [cited 31 Mar 2022]. https://products.sanofi.us/synviscone/synviscone.html

[19] Hill J, Alford DP. Prescription Medication Misuse. Semin Neurol. 2018;38: 654–664. doi: 10.1055/s-0038-1673691 30522141

[20] Birnbaum HG, White AG, Schiller M, Waldman T, Cleveland JM, Roland CL. Societal costs of prescription opioid abuse, dependence, and misuse in the United States. Pain Med. 2011;12: 657–667. doi: 10.1111/j.1526-4637.2011.01075.x 21392250

[21] Reinhart M, Scarpati LM, Kirson NY, Patton C, Shak N, Erensen JG. The Economic Burden of Abuse of Prescription Opioids: A Systematic Literature Review from 2012 to 2017. Appl Health Econ Health Policy. 2018;16: 609–632. doi: 10.1007/s40258-018-0402-x 30027533 PMC6132448

[22] Oderda GM, Lake J, Rüdell K, Roland CL, Masters ET. Economic Burden of Prescription Opioid Misuse and Abuse: A Systematic Review. J Pain Palliat Care Pharmacother. 2015;29: 388–400. doi: 10.3109/15360288.2015.1101641 26654413

[23] Strassels SA. Economic burden of prescription opioid misuse and abuse. J Manag Care Pharm. 2009;15: 556–562. doi: 10.18553/jmcp.2009.15.7.556 19739878 PMC10437678

[24] National Institute on Drug Abuse. Is drug addiction treatment worth its cost? In: National Institute on Drug Abuse [Internet]. — [cited 15 Sep 2020]. https://www.drugabuse.gov/publications/principles-drug-addiction-treatment-research-based-guide-third-edition/frequently-asked-questions/drug-addiction-treatment-worth-its-cost

[25] Quincho-Lopez A, Benites-Ibarra CA, Hilario-Gomez MM, Quijano-Escate R, Taype-Rondan A. Self-medication practices to prevent or manage COVID-19: A systematic review. PLoS One. 2021;16: e0259317. doi: 10.1371/journal.pone.0259317 34727126 PMC8562851

[26] Sadio AJ, Gbeasor-Komlanvi FA, Konu RY, Bakoubayi AW, Tchankoni MK, Bitty-Anderson AM, et al. Assessment of self-medication practices in the context of the COVID-19 outbreak in Togo. BMC Public Health. 2021;21: 58. doi: 10.1186/s12889-020-10145-1 33407321 PMC7787400

[27] Dorobisz K, Dorobisz T, Janczak D, Zatoński T. Doxycycline in the Coronavirus Disease 2019 Therapy. Ther Clin Risk Manag. 2021;17: 1023–1026. doi: 10.2147/TCRM.S314923 34584416 PMC8464303

[28] Butler CC, Yu L-M, Dorward J, Gbinigie O, Hayward G, Saville BR, et al. Doxycycline for community treatment of suspected COVID-19 in people at high risk of adverse outcomes in the UK (PRINCIPLE): a randomised, controlled, open-label, adaptive platform trial. The Lancet Respiratory Medicine. 2021;9: 1010–1020. doi: 10.1016/S2213-2600(21)00310-6 34329624 PMC8315758

[29] Growing use of home Covid-19 tests leaves health agencies in the dark about unreported cases. In: STAT [Internet]. 7 Dec 2021 [cited 31 Mar 2022]. https://www.statnews.com/2021/12/07/growing-use-of-home-covid19-tests-leaves-health-agencies-in-the-dark/

[30] Wu SL, Mertens AN, Crider YS, Nguyen A, Pokpongkiat NN, Djajadi S, et al. Substantial underestimation of SARS-CoV-2 infection in the United States. Nat Commun. 2020;11: 4507. doi: 10.1038/s41467-020-18272-4 32908126 PMC7481226

[31] Miech R, Johnston L, O’Malley PM, Keyes KM, Heard K. Prescription Opioids in Adolescence and Future Opioid Misuse. Pediatrics. 2015;136: e1169–e1177. doi: 10.1542/peds.2015-1364 26504126 PMC4834210

[32] Editorial. The Lancet Respiratory Medicine null. Fake medicines: fighting on all fronts. Lancet Respir Med. 2018;6: 315. doi: 10.1016/S2213-2600(18)30152-8 29653806

[33] Ivanova S, Ivanov K, Petkova E, Gueorgiev S, Kiradzhiyska D. Methods for detection of the misuse of “anti-oestrogens and aromatase inhibitors” in sport. Biomedical Research (0970-938X). 2017;28: 7157–7166.

[34] El Osta R, Almont T, Diligent C, Hubert N, Eschwège P, Hubert J. Anabolic steroids abuse and male infertility. Basic Clin Androl. 2016;26. doi: 10.1186/s12610-016-0029-4 26855782 PMC4744441

[35] Van Hout MC. Nod and wave: an Internet study of the codeine intoxication phenomenon. Int J Drug Policy. 2015;26: 67–77. doi: 10.1016/j.drugpo.2014.06.016 25052240

[36] Coloma PM, Becker B, Sturkenboom MCJM, van Mulligen EM, Kors JA. Evaluating Social Media Networks in Medicines Safety Surveillance: Two Case Studies. Drug Saf. 2015;38: 921–930. doi: 10.1007/s40264-015-0333-5 26242616 PMC4579253

[37] Wiśniewski M, Religioni U, Merks P. Community Pharmacies in Poland—The Journey from a Deregulated to a Strictly Regulated Market. IJERPH. 2020;17: 8751. doi: 10.3390/ijerph17238751 33255672 PMC7728088

[38] Merks P, Jakubowska M, Drelich E, Świeczkowski D, Bogusz J, Bilmin K, et al. The legal extension of the role of pharmacists in light of the COVID-19 global pandemic. Research in Social and Administrative Pharmacy. 2021;17: 1807–1812. doi: 10.1016/j.sapharm.2020.05.033 32546449 PMC7289723

[39] Barrett R. Evaluation of community pharmacists’ readiness to implement the Falsified Medicines Directive (Directive 2011/62/EC): an English cross-sectional survey with geospatial analysis. BMJ Open. 2020;10: e033405. doi: 10.1136/bmjopen-2019-033405 31924640 PMC6955531

[40] Zaprutko T, Kopciuch D, Paczkowska A, Sawicka D, Stachowiak Z, Bogdaniec P, et al. Access to vaccination in the Greater Poland (Poland). Acta Poloniae Pharmaceutica—Drug Research. 2019;76: 195–201. doi: 10.32383/appdr/99682

[41] Barrett R, Al-Mousawi HA. Development and initial validation of a postal survey evaluation of community pharmacists’ opinion regarding falsified (counterfeit) medicines in Hampshire (UK). Journal of Pharmacy and Pharmacognosy Research. 2018;6: 242–249.

[42] Mackey TK, Nayyar G. A review of existing and emerging digital technologies to combat the global trade in fake medicines. Expert Opin Drug Saf. 2017;16: 587–602. doi: 10.1080/14740338.2017.1313227 28349715

